# Postoperative Bleeding Following Preoperative Clopidogrel Administration in Patients with Haemoglobin Level Above 110 g/L Undergoing Urgent CABG

**DOI:** 10.21470/1678-9741-2017-0083

**Published:** 2018

**Authors:** Sasa Milan Kacar, Aleksandar Mikic, Mirjana Božidar Kačar

**Affiliations:** 1 Clinical Centers of Serbia - Clinic for Cardiac Surgery, Beograd, Serbia.

**Keywords:** Acute coronary syndrome, Hemorrhage/prevention & control, Coronary artery bypass, Platelet aggregation inhibitors, Blood platelets, Treatment outcome, Mortality, Morbidity, Survival

## Abstract

**Introduction:**

Patients with acute coronary syndrome usually receive dual antiplatelet therapy (DAPT) (usually clopidogrel + aspirin) prior to coronary catheterization, and approximately 10% of these patients require coronary artery bypass grafting (CABG). DAPT has favorable effects on prevention of thrombus formation, but it can have deleterious effects on surgical hemostasis. Anaemia, if present, gives additional risk to such patients. The aim of this study was to examine if DAPT affects postoperative bleeding in patients with haemoglobin levels above 110 g/L, who underwent urgent or emergent CABG, less than five days after stopping DAPT therapy.

**Methods:**

Data were collected prospectively on 122 CABG patients, operated by a surgical team from March 2008 to August 2013. Patients were stratified into two groups: group 1 received DAPT within 5 days of CABG (n=65), and group 2 where DAPT was discontinued for more than 5 days prior to CABG (n=57). All patients were diagnosed with acute coronary syndrome preoperatively, and all of them had haemoglobin levels above 110 g/L. Patients who needed reoperation, combined procedures, or off-pump revascularization were excluded.

**Results:**

There was no hospital mortality. Mean chest tube losses after the surgical revascularization did not differ significantly, but group 1 received a higher quantity of transfused red blood cells and platelets.

**Conclusion:**

Urgent and emergent surgical revascularization using extracorporeal circulation in patients with acute coronary syndrome whose preoperative haemoglobin levels are above 110 g/L is a safe and effective procedure. We suggest that, where indicative, one may perform CABG in less than 5 days after the clopidogrel discontinuation.

**Table:** 

Abbreviations, acronyms & symbols	
ACS	= Acute coronary syndrome
ACT	= Activated clotting time
ANOVA	= Analysis of variance
CABG	= Coronary artery bypass grafting
CPB	= Cardiopulmonary bypass
CURE	= Clopidogrel in Unstable angina to prevent Recurrent Events
DAPT	= Dual antiplatelet therapy
FFP	= Fresh frozen plasma
MACCE	= Major adverse cardiac and cerebrovascular events
PCI	= Percutaneous coronary intervention
RBC	= Red blood cells

## INTRODUCTION

Ischemic heart disease is the most prevalent cause of death in Serbia; representing 57% of total mortality. Acute coronary syndrome (ACS) is one of the most dramatic subtypes of ischemic heart disease and represents an important challenge. Dual antiplatelet therapy (DAPT), mostly with acetylsalicylic acid and clopidogrel (P2Y12 receptor inhibitor), has become the basis of ACS therapy. Indeed, it is well established that DAPT in ACS significantly decreases the incidence of new thrombotic and ischemic events^[1-4]^.

ACS patients rarely demand coronary artery bypass grafting (CABG). However, up to 10% of these patients may present with clinical findings requiring urgent or emergent coronary surgery^[5]^. Noteworthy these patients constitute a surgical challenge due to the fact that majority of them received DAPT prior to coronary angiography, as well as taking into account their inherent hemodynamic instability and potential unknown or unrecognized comorbidity^[6-8]^. Furthermore, there is an elevated surgical risk due to the deleterious effects of DAPT on hemostasis during and after CABG^[5]^.

Before CABG one must take into account the deterioration of platelet function by the preoperative DAPT usage, as well as the impairment of platelet function due to cardiopulmonary bypass (CPB) *per se.* Up to date, the management of preoperative antiplatelet therapy in CABG differs considerably among surgeons. In this respect, there is conflict between guidelines and prejudices regarding the preoperative aspirin usage. Indeed, in one hand, published data suggest that aspirin treatment should be held for 7-10 days preoperatively in patients undergoing elective CABG^[9]^, whereas more recent reports indicate that patients with coronary artery disease referred to CABG should continue aspirin treatment until the surgical procedure^[10]^. Likewise, clopidogrel usage was established to improve outcome after ACS by reducing early stent failure and diminishing allcause mortality and cardiovascular mortality^[11]^.

However, numerous studies have shown that CABG performed within 5 days of clopidogrel administration is associated with increased postoperative bleeding, reoperation for bleeding and prolonged hospital stay. Indeed, current guidelines recommend to postpone CABG if possible for 5 or more days after clopidogrel withdrawal^[12]^. On the other hand, withdrawing all DAPT for more than 5 days before surgery can increase the risk of fatal and non-fatal ischemic events, as well as increasing the mortality of patients with ACS^[11]^. Indeed, although mediastinal bleeding after CABG is not uncommon, it has an important influence on mortality and morbidity^[9]^.

Sabatine et al.^[13]^ showed that preoperative anemia, if present, is an independent predictor of major adverse cardiovascular events in patients with ACS. In patients with ACS without ST elevation, the risk for cardiovascular death, myocardial infarction, or recurrent ischemia increases if hemoglobin levels are below 110 g/L. It is the same in patients with ST elevation myocardial infarction, but when hemoglobin levels fall below 140 g/L^[13]^.

Low hemoglobin level is a strong predictor for postoperative need for transfusion, especially in CABG patients operated using extracorporeal circulation^[14]^.

The aim of this study was to examine if DAPT affects postoperative bleeding in patients with haemoglobin levels above 110 g/L, who underwent urgent or emergent CABG, less than five days after stopping DAPT therapy.

## METHODS

From March 2008 to June 2012, 122 patients with ACS underwent CABG using CPB, in the first 10 days after the coronary catheterization, by the same surgical team at the Institute for Cardiovascular diseases in Sremska Kamenica (Novi Sad). All patients received DAPT before coronary catheterization. All surgeries were described as urgent or emergent. Exclusion criteria were elective patients, combined procedures, reoperations, offpump revascularizations and preoperative anemia (hemoglobin levels less than 110 g/L). The following data were recorded: standard demographics, comorbidity and routine intraoperative and postoperative parameters, including blood loss and transfusion requirements in the first 48 hours postoperatively. The primary outcome was blood loss in the first 48 hours along with transfusion requirements, and the need for reoperation for bleeding. The secondary outcome was in-hospital mortality and hospital length of stay.

The patients were classified into two groups: 65 patients operated within 4 days (group 1), and specifically (day 0, 26 patients; day 1, 18 patients; day 2, 10 patients; day 3, 10 patients; day 4, 1 patient), whereas 57 patients operated from 5^th^ to 10^th^ day after clopidogrel discontinuation (group 2).

All operations were performed in normothermia, using CPB. The standard circuit was primed with a crystalloid solution. We were using roller pump in all patients. Standard heparinization protocol was applied (300 U/kg). Tranexamic acid was given to all patients in dosage of 30 mg/kg. Cold crystalloid cardioplegia (10-15 mL/kg) was used for myocardial protection. After weaning from CPB, heparin was neutralized with protamine sulfate (1 mg/100 U heparin).

The clinical criterion for platelet and fresh frozen plasma (FFP) transfusion was excessive bleeding despite the fact that the activated clotting time (ACT) value was normal and that there was no recognized surgical bleeding. The criteria for erythrocyte transfusion were a fall in hemoglobin below 90 g/L. The indication for re-exploration was bleeding over 400 mL during the first hour, 300 mL for 2-3 hours, or more than 200 mL/h during the next four hours despite the normal value of ACT. The late re-exploration was indicated when there was an echocardiographically proven pericardial effusion greater than 1.5 cm, or cardiac tamponade.

### Statistical Analysis

Statistical analysis was performed using SPSS 17.0 statistical software. Continuous variables were evaluated using analysis of variance (ANOVA); categorical variables were evaluated using χ2 analysis or Fisher's exact test. Multivariable models were created using logistic regression techniques for dichotomous outcomes and linear regression techniques for continuous outcomes.

## RESULTS

Baseline characteristics of all patients are presented in Table 1. There were no statistically significant differences in age, gender, presence of diabetes, renal failure, peripheral vascular disease, chronic obstructive pulmonary disease, neurologic disorders or gastrointestinal disease between the two groups.

**Table 1:** Baseline characteristics of patients.

		Group 1	Group 2	*P*-value
Gender	Men	39 (68.4%)	44 (66.7%)	Non-significant
	Women	18 (31.6%)	22 (33.3%)	
Age	Mean age ± SD	61.8±8.1	60.8±9.6	Non-significant
	Over 65 years old	21 (36.8%)	25 (37.9%)	
Diabetes		9 (15.8%)	12 (18.2%)	Non-significant
Renal failure		3 (5.3%)	1 (1.5%)	Non-significant
Peripheral vascular disease		11 (19.3%)	13 (19.7%)	Non-significant
Chronic obstructive pulmonary disease		18 (31.6%)	22 (33.3%)	Non-significant
Neurologic disorder		4 (7%)	7 (10.6%)	Non-significant
Gastrointestinal disease		1 (1.8%)	5 (7.6%)	Non-significant

Intraoperative data are presented in Table 2. There were no statistically significant differences in the respective variables such as number of grafts, CPB time or aortic cross-clamping time.

**Table 2:** Intraoperative characteristics of patients.

Intraoperative characteristics	Group 1	Group 2	*P*-value
Patients with more than 3 grafts	51 (77.3%)	40 (70.2%)	0.371
CPB time (min) (mean ± SD)	63.7±20.7	59.5±18.8	0.251
Aortic cross-clamping time (min)	37.8±12.6	35.7±11.9	0.353

CPB=cardiopulmonary bypass; SD=standard deviation

Intraoperative characteristics of patients are presented in Table 3. Interestingly, there was a surprisingly significant increase in the number of patients whose postoperative chest drainage was more than 500 mL in the control group (group 2) as compared to the group 1 - 62.1% of patients in group 2, and 40.4% in group 1 (*P*=0.019). Mean chest tube losses for 48 hours after surgical revascularization were 0.65 L in group 1 and 0.68 L in group 2 (Table 3). We show that patients in group 1 received a greater statistically significant quantity of red blood cells (RBC), platelets and cryoprecipitate compared to group 2 patients. The mean quantities of transfused RBC were 0.64 L and 0.47 L, respectively. There was no significant difference in the quantity of received FFP. Thus, the mean quantity of FFP given to the group 1 was 0.27 L, and to group 2 was 0.2 L. Platelets have been given to 12 patients in group 1, and to 1 patient in group 2.

**Table 3:** Postoperative chest drainage.

Postoperative chest drainage	Group 1	Group 2	*P*-value
Mean chest drainage	0.65 L	0.68 L	Non-significant
Chest drainage more than 500 mL	40.4	62.%	0.019

There was no in-hospital and 30-day mortality observed in any group. Two patients underwent reexploration for excessive bleeding, one in each group. The median length of stay was 13 days for group 1 and 19 days for group 2 patients.

## DISCUSSION

Impaired platelet function affects hemostasis, which may result in excessive postoperative blood loss and increased surgical complications and mortality. The etiology of excessive postoperative blood loss is multifactorial, including small body size, female gender and concomitant procedures^[6,7]^. In many patients with ACS, DAPT is not the only therapy that can influence surgical bleeding. Preoperative anticoagulants can affect hemostasis to a greater extent, and can be an important factor in the etiology of excessive blood loss. Moreover, about 3% of all CABG patients require reoperation for bleeding, which is followed by significant morbidity and mortality^[7,8]^. Under these circumstances, disseminated intravascular coagulopathy, hemodynamic instability and death are not uncommon. Furthermore, excessive blood loss is associated with increased blood and platelet transfusions, as well as administration of coagulation factors.

Anemia, if present, increases the need for postoperative transfusions. Anemia is also associated with increased mortality and postoperative morbidity in CABG patients^[15,16]^. That is the reason why we excluded patients with preoperative hemoglobin levels below 110 g/L in our study.

The results of the present study show that the use of clopidogrel within 5 days of CABG (in non-anemic patients) was not associated with increased postoperative blood losses, as well as with an increased number of re-explorations for postoperative bleeding. The preoperative use of clopidogrel, however, was associated with an increased number of RBC and platelet transfusions.

Over the last years, different clinical studies on DAPT in ACS have shown conflicting results. Differences in these findings and conclusions can be explained by variations in surgical indications, presence of comorbidity, differences in cardiological, anesthesiologic and surgical skills, as well as differences in preoperative therapy. Indeed, many studies have shown that clopidogrel administration prior to surgical revascularization is followed by increased incidence of postoperative bleeding, transfusion requirements and re-explorations with prolonged hospital stay^[17-20]^.

Berger et al.^[19]^ compared two groups of patients in relatively large retrospective study. In the group of patients who received clopidogrel in less than 5 days prior to CABG, there was an increased incidence of bleeding, re-explorations and increased hospital stay. On the other hand, this group of patients had significantly increased incidence of preoperative myocardial infarction and cerebrovascular accidents, previous percutaneous coronary intervention (PCI) procedures and more urgent surgical revascularizations. There were no differences in mortality and major adverse cardiac and cerebrovascular events (MACCE)^[19]^. Furthermore, Yende et al.^[18]^ reported increased re-exploration rate in patients with DAPT within 5 days of surgical revascularization. The study by Hongo et al.^[17]^ demonstrated increased 24-hour chest tube drainage, transfusion requirements, and reoperation rate for bleeding of patients on clopidogrel within 7 days of operation. In summary, the authors of both studies were against routine DAPT administration before PCI because of the increased morbidity risk if emergent CABG was mandatory. In a small series of 55 patients receiving clopidogrel preoperatively, significantly increased blood losses and transfusion requirements were reported^[21]^. A subgroup analysis of the Clopidogrel in Unstable angina to prevent Recurrent Events trial (CURE) trial identified 912 patients who had discontinued clopidogrel within 5 days of CABG In this group of patients, there was an increased risk of minor bleeding complications and a trend towards an increased risk of major bleeding complications, defined as substantially disabling bleeding or requiring more than 2 units of packed RBC transfusion, was shown^[5,22]^. On the other hand, in a recent study by Chen et al.^[23]^, preoperative clopidogrel exposure was not associated with increased RBC transfusion. Furthermore, there are some studies that underline the influence of both surgical and institutional experience on the results^[24]^.

In our study, only one surgical team (same surgeon and anesthesiologist) operated all patients enrolled in a specialized institution. There were no elective patients, and the decision of a surgical timing was made according to the clinical status of the patient. When considering mortality and morbidity of patients enrolled, we can see that there was no mortality in both groups of patients, but that patients in group 2 had a longer postoperative hospital stay compared to patients in group 1. However, these observations were restricted to in-hospital mortality and 30-day mortality.

According to current guidelines, it is suggested to optimally refrain from using clopidogrel within at least 5 days prior to elective surgery to minimize the risks of postoperative coagulopathy; that the emergent patients should proceed immediately to surgery, while semi-urgent patients should be treated on a case-by-case basis^[25-27]^.

The cardiologist, anesthesiologist and the surgeon must balance the risk of further ischemic events against the risk of postoperative bleeding and, according to our institutional policy, we operate all unstable patients and wait for 5 or more days in patients who can be medically stabilized.

## CONCLUSION

Urgent and emergent surgical revascularization using CPB in patients with ACS who were on DAPT preoperatively (withdrawal period 1-4 days), whose hemoglobin level is not less than 110 g/L, is a safe and effective procedure. The results of the present study showed that preoperative use of clopidogrel did not increase the risk of postoperative bleeding and did not affect the in-hospital and 30-day mortality. Further prospective randomized studies in a larger number of patients with a longer follow-up are needed to draw a conclusion that would benefit this important and special subgroup of patients.

It should be noted that the particularity of the targeted patient group, in addition to the ongoing recommendations, requires an integrated patient-to-patient approach to determine *pro et contra* of early or emergent surgery despite an alleviated risk of bleeding and complications.

**Table:** 

Authors' roles & responsibilities	
SMK	Agreement to be accountable for all aspects of the work in ensuring that questions related to the accuracy or integrity of any part of the work are appropriately investigated and resolved; final approval of the version to be published
AM	Agreement to be accountable for all aspects of the work in ensuring that questions related to the accuracy or integrity of any part of the work are appropriately investigated and resolved; final approval of the version to be published
MBK	Agreement to be accountable for all aspects of the work in ensuring that questions related to the accuracy or integrity of any part of the work are appropriately investigated and resolved; final approval of the version to be published
